# Iron Metabolism Dysregulation and Cognitive Dysfunction in Pediatric Obesity: Is There a Connection?

**DOI:** 10.3390/nu7115458

**Published:** 2015-11-06

**Authors:** Anna Grandone, Pierluigi Marzuillo, Laura Perrone, Emanuele Miraglia del Giudice

**Affiliations:** Department of Woman, Child, General and Specialized Surgery, Second University of Naples Via De Crecchio 2–4, Naples 80138, Italy; pierluigi.marzuillo@gmail.com (P.M.); laura.perrone@unina2.it (L.P.); emanuele.miraglia@unina2.it (E.M.D.G.)

**Keywords:** obesity, iron deficiency, cognitive dysfunction, hepcidin, children

## Abstract

Obesity and iron deficiency (ID) are two of the most common nutritional disorders in the world. In children both conditions deserve particular attention. Several studies revealed an association between obesity and iron deficiency in children and, in some cases, a reduced response to oral supplementation. The connecting mechanism, however, is not completely known. This review is focused on: (1) iron deficiency in obese children and the role of hepcidin in the connection between body fat and poor iron status; (2) iron status and consequences on health, in particular on cognitive function; (3) cognitive function and obesity; (4) suggestion of a possible link between cognitive dysfunction and ID in pediatric obesity; and implications for therapy and future research.

## 1. Introduction

Obesity and iron deficiency (ID) are two of the most common nutritional disorders in the world. In children, both conditions deserve particular attention to avoid future complications [1]. Indeed, the prevalence of childhood obesity has increased epidemically worldwide with important consequences for the health of children, increasing the risks for type 2 diabetes, cardiovascular events throughout life, and liver damage [2,3,4]. On the other hand, iron deficiency is an important health problem both in developed and developing countries, increasing the risk of anemia and impaired cognition, behavior, and motor skills [5,6,7].

Several studies revealed an association between obesity and iron deficiency in children [8,9,10,11,12,13] and in some cases a reduced response to oral iron supplementation [14]. The connecting mechanism, however, is not completely known [15].

Considering that iron deficiency seems to impair cognitive function and exercise performance [5], and that obese children present with poorer cognitive functions [16], we hypothesize that obesity-related ID may impair brain development in children.

This review is focused on: (1) iron deficiency in obese children and the role of hepcidin in the connection between body fat and poor iron status; (2) iron status and consequence on health, in particular on cognitive function; (3) cognitive function and obesity; (4) suggestion of a possible link between cognitive dysfunction and ID in pediatric obesity; and implications for therapy and future research.

## 2. Iron Homeostasis

Iron is an essential element for all living organisms. It is a key component of oxygen-carrying proteins playing a vital role in cellular metabolism and cell growth and differentiation [17]. Systemic iron is tightly regulated to allow for a balanced and stable concentration of iron levels both in plasma and the extracellular fluid. Whole body iron homeostasis results from maintaining the major iron flows [17]. Iron homeostasis results from the interplay between plasmatic (hepcidin/ferroportin-1 mediated) and cellular (iron regulatory proteins (IRPs)/iron responsive element (IRE) mediated) body iron sensing systems. Systemic iron regulation occurs through three major mechanisms: (i) absorption of dietary iron via the enterocytes of the proximal duodenum; (ii) release of stored iron from the hepatocytes; and (iii) release of stored iron from reticuloendothelial macrophages [18]. Within the cell, iron levels are sensed by IRPs. When cytoplasmic iron is low, IRPs bind to IRE sequences in the 3′ region of the mRNA of iron-regulated proteins, including the transferrin receptor and ferritin with consequent increased protein synthesis [18]. Conversely, when cytoplasmic iron is adequate, the IRPs bind to the IRE in the 5′ region of the same mRNA with consequent decreased protein production [18].

## 3. Hepcidin, Master Regulator of Systemic Iron Homeostasis

Hepcidin is a small peptide hormone that functions as both the homeostatic regulator of systemic iron metabolism and mediator of host defense and inflammation, and is measurable in human urine, plasma, and serum [17,18,19,20,21]. Sensing of circulating iron and iron store levels is thought to occur in the liver, which is the primary site of hepcidin production and secretion [17,22].

Hepcidin is also produced to a lesser degree in the adipose tissue, heart, placenta, and kidneys although *in vivo* secretion and contribution to circulating levels from these sites is currently unknown [18,20]. Hepcidin is a major regulator of iron availability for erythropoiesis. It inhibits iron intestinal absorption and reduces the flux of iron from splenic and hepatic macrophages, as it binds to the cellular iron export channel ferroportin-1, present in enterocytes, hepatocytes and macrophages, promoting its internalization and degradation [23]. The hepatic production of hepcidin is up-regulated by proinflammatory cytokines, such as interleukin (IL)-6, bone morphogenetic proteins and iron overload; it is down-regulated by iron deficiency, hypoxia and ineffective erythropoiesis [24]. Higher hepcidin levels are observed in inflammatory anaemia, a pathology associated with reduced iron bioavailability and mobilization from stores and clinically characterized by decreased iron blood levels and increased cellular iron stores [25]. Some hepcidin production and secretion has been documented in adipose tissue explants, but *in vivo* studies do not confirm these findings. Thus, more studies are needed to better clarify the role of adipose tissue in systemic hepcidin levels [26].

## 4. Iron Deficiency in Obese Children

**Figure 1:**
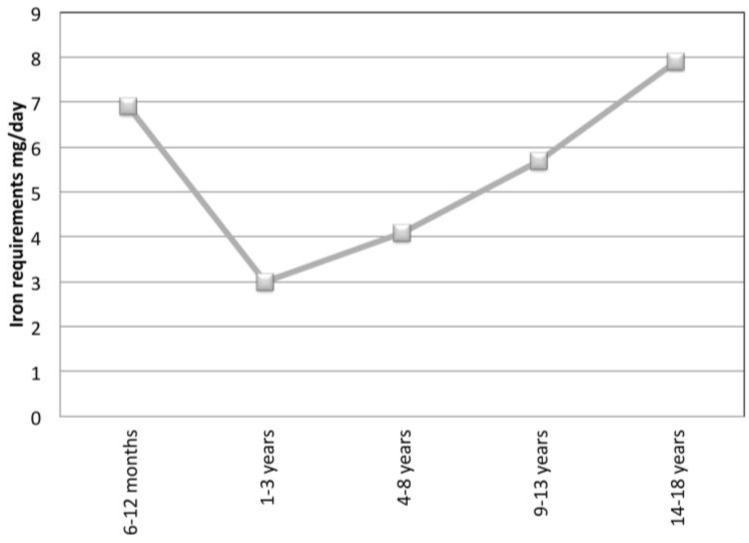
Iron requirements (mg/day) from 6 months to 18 years (data from Food and Nutrition Board, Institute of Medicine, National Academies).

Children, and in particular infants and adolescents, are a population at risk to develop iron deficiency, probably due to the increased demand (Figure 1). The iron requirement is reduced from sixth months to three years. Then, it gradually increases reaching the max values during pubertal development. The first report of potential connection between iron status and obesity appeared over 40 years ago [9]. This report described lower serum iron concentrations in obese adolescents compared to normal weight controls. Since then many other studies have reported that obese children, and in particular adolescents, are at risk for ID both from the US and transition countries [10,11,12,13].

At least three potential mechanisms of hypoferremia in obesity have been proposed and include; (i) nutritional iron deficiency (even if data are contrasting) [27,28,29]; (ii) elevated blood volume as a function of increased adipose tissue mass leading to enhanced iron requirements [27,30]; and (iii) systemic low grade inflammation typical of obesity [27,31].

## 5. Proposed Mechanisms Linking Obesity and Iron Deficiency: The Role of Hepcidin

**Figure 2:**
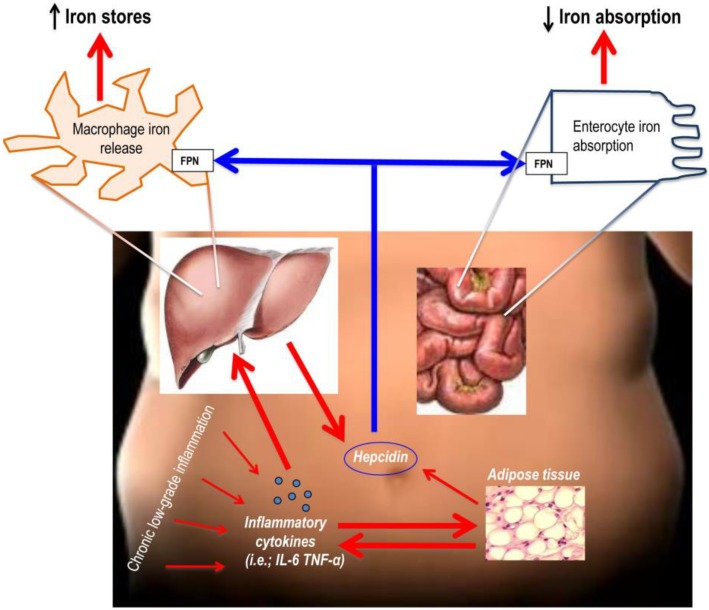
The role of hepcidin in the connection between adiposity and poor iron status.

The chronic low-grade inflammation typical of the obesity leads to inflammatory cytokines production with consequent stimulation of hepatic hepcidin production. Also the adipose tissue appears to produce minor levels of hepcidin. Then, hepcidin acts on the enterocytes resulting in reduced iron absorption, and on splenic and hepatic macrophages resulting in less iron release and increased iron stores (red arrows stimulation, blue arrows inhibition) (Figure 2).

Several researchers have demonstrated that serum hepcidin is significantly elevated in obese compared to lean women and children [14,32]. One study reported that overweight children have higher circulating hepcidin and poorer iron status, despite similar dietary iron intake, when compared to normal weight children [14]. In another study, overweight children were found to have higher serum hepcidin and lower serum iron and transferrin saturation (TSAT) compared to normal weight children [32]. Further, serum hepcidin was inversely correlated with iron absorption and positively with serum leptin, an adipokine found to be elevated in obesity [32]. Also, in a more recent study, a strong positive relationship between body mass index (BMI) SDS and circulating hepcidin was observed. In this same cohort, IL-6 levels were found to be positively correlated with hepcidin [33]. These studies largely concluded that ID in obesity is likely due to hepcidin-mediated reduction in iron absorption and/or sequestration, and that increased serum hepcidin levels may be due in part to subcutaneous and visceral adipose tissue secretion of the protein as well as increased liver hepcidin production and secretion mediated by inflammation [14,32].

In addition to hepcidin, lipocalin-2 production may be important in inflammation-induced obesity-related ID [27]. Lipocalin-2 is a protein produced by liver, pancreas and adipose tissue in response to inflammation and plays a role in innate immunity by sequestrating iron and subsequently limiting iron availability for pathogenic bacteria [27]. There is evidence that obesity is characterized by increased circulating levels [34] and increased adipocyte expression of lipocalin-2 [35,36]. However, these data have not been confirmed in pediatric obese population [37].

Thus, inflammatory mediated hepcidin and potentially lipocalin-2 could have a negative effect on iron absorption and explain, in part, the ID observed in obese pediatric populations.

## 6. Iron Deficiency and Cognition

Iron deficiency is defined as a condition in which there is no mobilizable iron stores and in which signs of a compromised supply of iron to tissues is detected [5]. Consequences of iron deficiency in pediatric age can be particularly important. In fact there is a consensus that ID, without, concurrent anemia, has a negative impact on cognition, behavior, and motor skills that can persist in the long-term [5]. Specifically, cognitive disturbances associated with ID are related to attention span, intelligence, and sensory perception [5]. In fact, patients with chronic ID present with lower scores on language, environmental sound perception, and motor measures when compared with children with normal nutritional iron status [38,39]. Further, ID is associated with alterations in many metabolic processes that may impact brain functioning (e.g., mitochondria electron transport, neurotransmitter synthesis and degradation, protein synthesis, and organogenesis) [5]. It is likely that ID impacts hippocampal function [6] as result of mitochondrial damage [39]. In addition, changes in brain dopamine metabolism are presumed to occur in response to ID [40,41], as well as altered serotonergic neurotransmission [42] and dopamine receptor function [43]. Moreover, Lozoff [44] has reported that alterations in the mesolimbic pathway, positive affect, and inherent reward might support the explanation for the altered socio-emotional behavior that has been described in children with ID. Therefore, iron status appears to be a crucial determining factor of cognitive functioning in children and its disruption implies significant consequences on child brain health.

## 7. Obesity and Cognitive Function

Obesity in youth is associated with poorer cognitive functioning as measured by neurocognitive tasks and self-report measures [16]. In cross-sectional studies, it has been shown that obese children present with lower global functioning, cognitive ability, and full scale IQ compared with normal weight children [45,46]. Moreover, examining a sample of overweight and sedentary youths, BMI *z*-score, waist circumference, body fat, visceral fat and abdominal fat were all negatively correlated with cognitive ability [47]. There is also strong and consistent evidence supporting a relationship between obesity and deficits in visuospatial skills [45,48], and motor skills, specifically: coordination, balance, strength, and fine and gross motor skills [49,50,51,52,53,54,55,56,57,58,59,60,61]. Others [49,54] have shown that excess body weight is inversely associated with attention and concentration in obese compared to normal weight children. It has also been reported that executive function is affected by overweight [51,52] with an improvement observed with weight loss [53]. Moreover obesity-related behaviors such as increased food intake, disinhibited eating, sedentary behavior, and lower physical activity are generally related to executive dysfunction, poorer motor skills, and lower academic achievement [16,55]. The precise mechanisms linking obesity with lower cognition functioning remains unclear. Researchers have considered ID and obesity separately as predisposing factors for impaired cognitive function [5,6,39,40,41,42,43,44,56,57,58,59]. However, we speculate that inflammation-mediated obesity related ID could be significant factor associating obesity with poorer cognitive functioning. Elevated hepcidin could be the key factor causing ID in obese children predisposing this group to cognitive impairment.

## 8. Clinical Implications and Future Directions

Although more research is necessary to understand the connection between obesity-related ID and cognition in children, assessing the iron status of obese children is advisable. However, treating ID in obese individuals including children poses a challenge. Both Sanad *et al.* [60] and Zimmermann *et al.* [61] reported that oral iron supplementation therapy was not efficacious for replacing iron stores associated for obese children. Both studies observed that serum hepcidin was associated with hypoferremia and poorer response to oral iron therapy [60,61]. Thus, routine clinical practice of providing oral iron supplementation to ID individuals may not be effective for obese individuals.

Amato *et al.* [62] reported a significant decrease in serum hepcidin, increased Fe absorption, and improved Fe status following weight loss in obese children. Thus, weight loss could be the first step in the management of ID in obese children [62]. Similarly, Gong *et al.* demonstrated in a cohort of obese children that weight-loss was associated with improved iron and inflammatory status [63].

Therefore, oral Fe supplementation therapy combined with weight loss may be effective for correcting ID in obese children. Randomized-controlled trials evaluating usual care (oral iron supplementation), weight loss, *versus* weight loss and oral Fe administration could give useful information in delineating the appropriate therapy to treat ID in obese children.

Moreover, evaluation of cognitive functioning in the context of such trials may provide important information regarding the role of concurrent ID and obesity in precipitating cognitive dysfunction in obese children. In fact it would be important to follow different aspects of cognitive functioning in obese children in relation to ID and ID therapy, taking into account also socioeconomic factors (*i.e.*, the education levels of parents and employment), grade of obesity and age at its onset.

## 9. Conclusions

Both ID and obesity have been independently associated with poor cognitive function, but at the present time the pathophysiological mechanism(s) linking obesity, ID and cognitive dysfunction is unknown. The interaction between ID and obesity in determining cognitive dysfunction could be driven by elevated hepcidin and reduced iron bioavailability in obese children. Pediatricians should bear in mind the potential effect of obesity-related ID on cognition in obese children and the need to evaluate both iron status and the presence of cognitive dysfunction. Although additional research is necessary, clinicians must be conscious that the best treatment for obesity-related ID may not be of oral iron supplementation alone but instead weight loss alone or weight loss plus iron supplementation may improve iron status and ultimately cognitive functioning of obese children.
